# Intercondylar Route of Prosthetic Infragenicular Femoropopliteal Bypass Has Better Primary, Assisted, and Secondary Patency but Not Limb Salvage Rate Compared to the Medial Route

**DOI:** 10.1155/2016/1256414

**Published:** 2016-09-07

**Authors:** Tomas Grus, Lukas Lambert, Rohan Banerjee, Gabriela Grusova, Vilem Rohn, Tomas Vidim, Petr Mitas

**Affiliations:** ^1^Department of Cardiovascular Surgery, First Faculty of Medicine, Charles University in Prague and General University Hospital in Prague, Prague, Czech Republic; ^2^Department of Radiology, First Faculty of Medicine, Charles University in Prague and General University Hospital in Prague, Prague, Czech Republic; ^3^Fourth Department of Medicine, First Faculty of Medicine, Charles University in Prague and General University Hospital in Prague, Prague, Czech Republic; ^4^Department of Cardiovascular Surgery, Second Faculty of Medicine, Charles University in Prague, Prague, Czech Republic

## Abstract

*Aim*. To compare the differences between medial and intercondylar infragenicular femoropopliteal prosthetic bypasses in terms of their midterm patency and limb salvage rates.* Methods*. Ninety-three consecutive patients with peripheral arterial disease who underwent a simple distal femoropopliteal bypass using a reinforced polytetrafluorethylene graft were included in this retrospective study. The bypass was constructed in the intercondylar route in 52 of the patients (group A) and in 41 in the medial route (group B).* Results*. Median observation time of the patients was 12.7 (IQR 4.6–18.5) months. There were 22 and 24 interventional or surgical procedures (angioplasty, stenting, thrombolysis, thrombectomy, or correction of the anastomosis) performed to restore patency of the reconstruction in groups A and B, respectively (*p* = 0.14). The 20-month primary, assisted, and secondary patency rates and limb salvage rates were 57%, 57%, 81%, and 80% in group A compared to 21%, 23%, 55%, and 82% in group B (*p* = 0.0012, 0.0052, 0.022, and 0.44, resp.).* Conclusion*. Despite better primary, assisted, and secondary patency rates in patients with a prosthetic infragenicular femoropopliteal bypass embedded in the intercondylar fossa compared to patients with the medial approach, there is no benefit in terms of the limb salvage rate and the number of interventions required to maintain patency of the reconstruction.

## 1. Introduction

Femoropopliteal bypass surgery has been, for a long time, the most common infrainguinal reconstruction procedure in vascular surgery. Although the saphenous vein is usually the preferred conduit due to its superior patency rates, in around 20–40% of patients, it is simply not available for bypass in sufficient quality or length [1, 2]. Previous studies comparing different types of prostheses and adaptations of the distal anastomosis of below-knee reconstructions showed a wide range of primary patency rates between 24% and 83% during the first two or three postoperative years [2–6].

In the infragenicular femoropopliteal bypass, the distal part of the graft can be either embedded subcutaneously on the medial side of the knee or placed dorsally between the femoral condyles. However, little data exists to compare midterm performance of both approaches, which is influenced by numerous factors, including the outflow bed, type of the graft, anastomosis angle, and compliance mismatch [7–9].

In the present study, we compared the midterm primary, assisted, and secondary patency rates and limb salvage rates of the infragenicular femoropopliteal bypasses between patients with the intercondylar or medial course of the bypass.

## 2. Patients and Methods

This retrospective study was approved by the institutional review board (Ethical Committee of the General University Hospital in Prague) and informed consent was waived. The research was performed in accordance with the Declaration of Helsinki. Between July 2009 and September 2015, 93 consecutive patients with peripheral arterial disease stage IIb to stage IV according to the Fontaine classification who underwent a simple distal femoropopliteal bypass (i.e., without a cuff or patch) using a reinforced polytetrafluorethylene (PTFE) prosthesis were included in the study [10]. The bypass was constructed in the intercondylar route in 52 patients (group A) and in the medial route in 41 (group B). All patients had at least one patent crural artery, as depicted by preoperative digital subtraction angiography (DSA) or CT angiography. Patients' characteristics are listed in Table 1.

### 2.1. Surgical Technique

The surgical procedures were performed by three consultant vascular surgeons. In all the patients, a reinforced vascular PTFE prosthesis either VascuGraft SOFT (B. Braun Melsungen, Berlin, Germany) or fusion vascular graft (Maquet Holding, Rastatt, Germany) with a diameter from 6 to 8 mm to match the diameter of the popliteal artery was used (Table 2).

**Figure 1:**
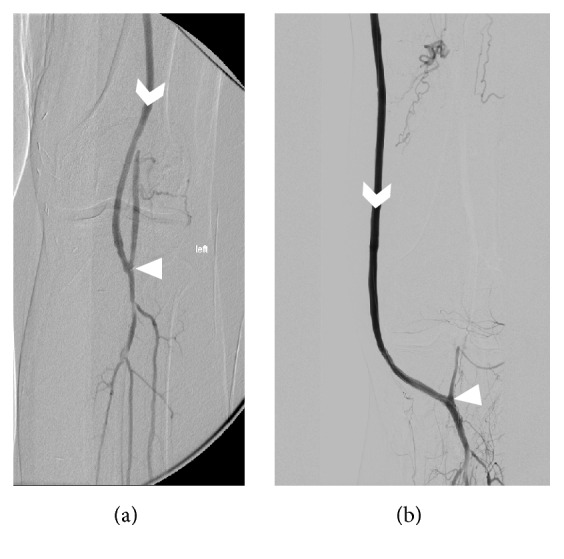
Digital subtraction angiography (DSA) of lower limbs shows intercondylar (a) and medial (b) route of an infragenicular femoropopliteal bypass. Arrowheads denote the anastomosis and chevrons show the flow direction in the bypass.

The proximal anastomosis was always connected to the common femoral artery and the distal anastomosis to the popliteal artery, below the knee joint space. If the subcutaneous layer on the medial side of the knee was sufficiently thick (estimated subjectively by surgical palpation), the bypass was routed there (group B), with subsequent diversion towards the popliteal artery below the knee (Figure 1). In the rest of the patients, the graft was embedded beneath the femoral fascia and turned towards the proximal part of the popliteal artery first and then later connected to its distal part (group A, Figure 1). We attempted to construct the distal anastomosis preferably with a smaller anastomosis angle to reduce adverse hemodynamics known to be responsible for intimal hyperplasia [8, 9].

### 2.2. Medication

For antimicrobial prophylaxis, four doses of 1.5 g ampicillin/sulbactam (Haupt Pharma Latina, Borgo San Michele, Italy) were given intravenously 8 hours apart. Postoperatively, all patients received acetylsalicylic acid (Anopyrin, Zentiva, Czech Republic, 100 mg daily) and low-molecular-weight heparin (nadroparin, 0.1 mL/10 kg, Aspen Pharma, Dublin, Ireland). After mobilization and removal of drains (usually from the 3rd-4th postoperative day), patients continued with dual antiplatelet therapy (acetylsalicylic acid 100 mg daily and clopidogrel 75 mg daily (Thrombex, Zentiva, Czech Republic)) only, except for those who had received anticoagulation therapy preoperatively and needed reintroduction of the oral anticoagulant treatment.

All patients were discharged with statin (at least 20 mg daily, most commonly atorvastatin) and dual antiplatelet therapy or, in case of anticoagulant treatment, with warfarin (Orion Corp., Espoo, Finland) and acetylsalicylic acid (100 mg daily).

### 2.3. Follow-Up

Follow-up examinations were scheduled approximately 1, 3, 6, 12, and 24 months postoperatively, unless clinical problems occurred. Apart from clinical examination, patency of the reconstruction was examined by ultrasound. CT angiography or digital subtraction angiography was indicated if the patient's complaints or clinical examination suggested stenosis or occlusion. In case of restenosis or occlusion, angioplasty, stenting, thrombolysis, thrombectomy, or correction of the anastomosis was performed according to interdisciplinary consent based on the patient's complaints and imaging findings. During follow-up, primary, assisted, and secondary patency limb salvage (preserved foot) and mortality were recorded.

### 2.4. Statistical Analysis

Statistical tests were performed using MedCalc ver. 12 (MedCalc Software bvba, Ostend, Belgium). To test for statistical significance, we used *t*-test, Mann-Whitney *U* test, or Fischer *F*-test as appropriate. Life tables were compared using the log rank test. A *p* value below 0.05 was considered significant.

## 3. Results

The mean duration of the operation and hospital stay was 120 (IQR 90–159) min and 6 days (IQR 5–10), respectively, in group A, compared to 130 (IQR 102–160) min (*p* = 0.38) and 6 days (IQR 4–7, *p* = 0.065) in group B (Table 2). No patient died perioperatively. During the first postoperative month, we recorded the following complications: early occlusion of the bypass (1 patient in group A and 2 patients in group B), pseudomembranous enterocolitis (1 patient in group A), wound dehiscence (2 patients in group A and 1 patient in group B), sepsis (1 patient in group A), mesenteric ischemia (1 patient in group A), and cardiac insufficiency (1 patient in group B).

**Figure 2:**
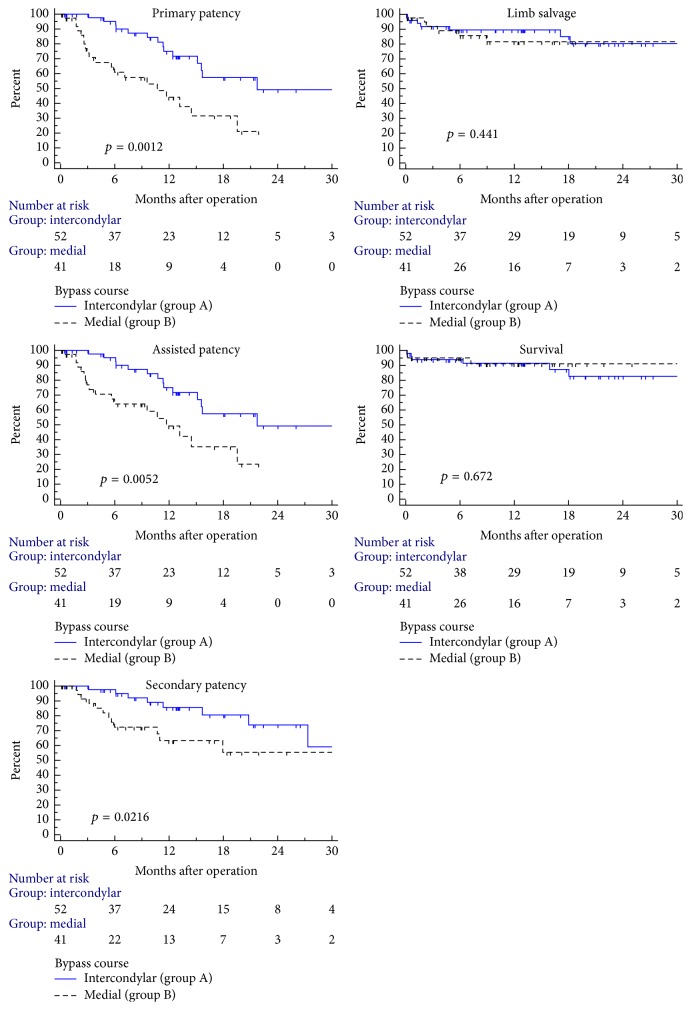
Kaplan-Meier plots of midterm primary, assisted, and secondary patency, limb salvage rates, and survival in patients with a distal prosthetic femoropopliteal bypass show that although femoropopliteal bypass in patients with an intercondylar course (group A) has better primary, assisted, and secondary patency rates compared to medial route (group B), the limb salvage rate and survival are similar.

Median observation time of the patients was 12.7 (IQR 4.6–18.5) months. The 20-month primary, assisted, and secondary patency rates and limb salvage rates were 57%, 57%, 81%, and 80%, respectively, in group A compared to 21%, 23%, 55%, and 82% in group B (*p* = 0.0012, 0.0052, 0.022, and 0.44, resp.). Comparison of primary, assisted, and secondary patency, limb salvage rates, and survival is shown in Figure 2. There were 22 and 24 interventional or surgical procedures (angioplasty, stenting, thrombolysis, thrombectomy, or correction of the anastomosis) performed to restore patency of the reconstruction in groups A and B, respectively (*p* = 0.14).

## 4. Discussion

Since the introduction of a prosthetic femoropopliteal bypass, there has been ongoing research into improvement of its patency, which has rarely matched that of reconstructions with autologous grafts [6, 11]. Major causes of femoropopliteal bypass failure vary in relation to the time from the operation: from technical error (and hypercoagulable states) early postoperatively to intimal hyperplasia in the first two years and then later to progression of atherosclerosis [12]. Intimal hyperplasia refers to thickening of the intima by proliferation of extracellular matrix and migration of smooth muscle cells as a response to endothelial injury and altered hemodynamics [8, 13, 14]. On a macroscopic level, changes in biomechanics of the reconstruction and hemodynamic parameters known to induce intimal hyperplasia (wall shear stress, stagnation point) have been described using numerical simulations and in vitro and in vivo models and even in patients [8, 14–16]. They can be influenced to a limited extent by the morphology of the anastomosis, including the anastomosis angle, ratio of diameter of the bypass and the target artery, and other modifications, such as venous cuffs and patches or precuffed grafts [3, 8, 17, 18].

In this study, we retrospectively evaluated midterm results of an infragenicular prosthetic femoropopliteal bypass with regard to its route in the knee region. The patency rates observed in group A were in accordance with a meta-analysis by Takagi et al. [4] that included predominantly above-knee bypasses as did other major randomized trials [19]. Tuchmann and Dinstl reported a patency of below-knee reinforced femoropopliteal bypass of 60% at 20 and 30 months after the operation [5]. A randomized trial published by Stonebridge and associates included analysis of below-knee reconstructions in a comparable group of patients with patency rates of 29% at 2 years and 19% at 3 years with improvement by 23% and 26%, respectively, when Miller cuff was used [20, 21]. Unfortunately, their study did not report the number of patent crural vessels, which substantially influences graft patency [22, 23]. Similar patency rate was found by Loh et al. with 49% patency rate at 3 years using precuffed PTFE grafts [24], by Kreienberg et al. with primary patency rate between 38% and 48% at 3 years [25], and by Donker et al. with 24% at three years [26]. Dorigo et al. showed better patency rate of 46% at 48 months even though 47% of the patients had only one patent run-off vessel [23] and Daenens et al. even showed a rate of 83% at two years (better than with autologous saphenous vein) in a sample containing greater proportion of claudicants using heparin-bonded ePTFE graft [6].

In theory, we assumed that a medial approach in group B would result in a less acute angle, because the graft needed to pass from the medial side of the knee to the dorsal side below the knee to attach to the popliteal artery [8, 9]. This may be one explanation as to why patency rates were better in group A. However, due to the retrospective nature of this study, we were unable to confirm this hypothesis because the angle was not measured during the operation. Nor can this be retrospectively assessed from the follow-up imaging due to the fact that the anastomosis undergoes remodeling postoperatively. Ultimately, there is no difference in the limb salvage rate, which is comparable to other studies, and therefore the clinical benefit of intercondylar route for a patient is limited [3, 4]. The survival of patients in this study is unnaturally high, probably due to the fact that a number of subjects have been lost to follow-up.

Although the medial route would be generally preferred, we believe that embedding a bypass medially if the subcutaneous layer is thin may carry an increased risk of bypass infection, compression against bony structures, and wound complications. Conversely, the intercondylar route possesses an increased risk of injury to the structures of the neurovascular bundle and the surrounding venous plexus in particular. In the infragenicular prosthetic femoropopliteal bypasses, we rarely use patch or cuff techniques because their advantage is controversial, with their construction additionally requiring about 15 minutes of operation time [27]. In our department, PTFE prostheses are preferred to knitted grafts, although their patency should be comparable [4, 28]. Promising improvement in graft patency is anticipated in new fish collagen coated low-flow knitted grafts, which are currently being tested in preclinical trials in our department.

The most important limitations of this study include a relatively short follow-up time, small number of patients in each group, and a lack of randomization because the study is retrospective. To overcome this limitation, a randomized trial with exclusion of patients with thin subcutaneous layer on the medial side of their knee would be necessary. Considering the event rate of 15% for limb amputation at 30 months, with hazard ratio of 1.5, such study would require randomization of 550 patients and a multicenter design to achieve 80% power with 5% significance level. To demonstrate the difference in the anastomosis angle, its perioperative measurement or early postoperative imaging would also be required.

## 5. Conclusion

Despite better primary, assisted, and secondary patency rates in patients with a prosthetic infragenicular femoropopliteal bypass embedded in the intercondylar fossa compared to patients with the medial approach, there is no benefit in terms of the limb salvage rate and the number of interventions required to maintain patency of the reconstruction.

## Figures and Tables

**Table 1:** Patients' characteristics.

Characteristics	Intercondylar bypass Group A	Medial bypass Group B	*p* value	Test
Number of patients	52	41		
Male gender	38	32	0.63	*F*-test
Age (years)	68 ± 8	69 ± 9	0.73	*t*-test

Coronary artery disease	2	15	0.67	*F*-test
Angina pectoris	6	3	0.73	*F*-test
Myocardial infarction	17	10	0.49	*F*-test
CABG	12	8	0.80	*F*-test
Atrial fibrillation	7	4	0.75	*F*-test
Stroke	10	8	1.0	*F*-test
Diabetes	22	18	1.0	*F*-test
Hypertension	44	33	0.78	*F*-test
Hyperlipidemia	37	35	0.14	*F*-test
Renal insufficiency	10	3	0.14	*F*-test
Smoker or ex-smoker	42	37	0.25	*F*-test
BMI	25.8 ± 3.0	27.5 ± 3.9	0.047	*t*-test

Preoperative medication				
Antiplatelet therapy	41	34	0.79	*F*-test
Anticoagulation	18	14	1.0	*F*-test
Statin	34	30	0.50	*F*-test

Fontaine classification			0.20	MW
IIB	4	5		
III	21	20		
IV	27	16		

TASC classification			0.43	MW
C	1	2		
D	51	39		

CABG: coronary artery bypass grafting; MW: Mann-Whitney test.

**Table 2:** Patients' data related to the operation.

Characteristics	Intercondylar bypass Group A	Medial bypass Group B	*p*	Test
Number of patients	52	41		

Side			0.093	*F*-test
Right	26	28		
Left	26	13		

Number of open run-off vessels			0.27	MW
1 artery	15	11		
2 arteries	26	15		
3 arteries	11	15		

Prosthesis diameter			0.16	MW
6 mm	11	12		
7 mm	37	29		
8 mm	4	0		

Prosthesis type			0.82	*F*-test
Fusion vascular graft	36	30		
VascuGraft SOFT	16	11		

Operation time (min)	120 (IQR 90–159)	130 (IQR 102–160)	0.38	MW
Hospital stay (days)	6 (IQR 5–10)	6 (IQR 4–6)	0.065	MW

IQR: interquartile range; MW: Mann-Whitney test.
